# Cytosolic and nuclear recognition of virus and viral evasion

**DOI:** 10.1186/s43556-021-00046-z

**Published:** 2021-10-10

**Authors:** Siji Li, Lili Cao, Zeming Zhang, Ming Kuang, Luoying Chen, Yingchi Zhao, Yujie Luo, Zhinan Yin, Fuping You

**Affiliations:** 1 Department of Clinical Laboratory, Ningbo First Hospital, Ningbo, Zhejiang, China; 2 Institute of Systems Biomedicine, Department of Immunology, School of Basic Medical Sciences, Beijing Key Laboratory of Tumor Systems Biology, Peking University Health Science Center, Beijing, China; 3 Zhuhai Institute of Translational Medicine, Zhuhai People’s Hospital Affiliated with Jinan University, Jinan University, Zhuhai, Guangdong China; 4 The Biomedical Translational Research Institute, Faculty of Medical Science, Jinan University, Guangzhou, Guangdong China

**Keywords:** Innate immunity, RNA sensor, DNA sensor, Viral evasion

## Abstract

The innate immune system is the first line of host defense, which responds rapidly to viral infection. Innate recognition of viruses is mediated by a set of pattern recognition receptors (PRRs) that sense viral genomic nucleic acids and/or replication intermediates. PRRs are mainly localized either to the endosomes, the plasma membrane or the cytoplasm. Recent evidence suggested that several proteins located in the nucleus could also act as viral sensors. In turn, these important elements are becoming the target for most viruses to evade host immune surveillance. In this review, we focus on the recent progress in the study of viral recognition and evasion.

## Introduction

Viruses, including influenza virus, Ebola virus, Zika virus, pose significant global public health threats. Immunization is essential for controlling and eliminating viral infections. The innate immune system provides nonspecific defense mechanisms against pathogens and comes into play immediately. Profound understanding of the relationship between viral recognition and viral evasion may provide new insights for the transmission and treatment of viral infectious diseases. In this review, we summarize recent discoveries about the cytosolic and nuclear viral nucleic acids sensing pathway. Besides, elaborate escape strategies for viruses at different steps of the signaling transduction are also included.

## Recognition of viral nucleic acids

Many kinds of pathogens experience intra-cellular stage during infection. After invasion, these pathogens utilize and hijack the host cellular environment and materials to facilitate their replication and proliferation. During this period, components of pathogens, such as nucleic acids and polysaccharides can be exposed. These components called the pathogen-associated molecular patterns (PAMPs) [1]. Host have evolved sophisticated mechanism to identify these foreign substances, which is known as the pattern recognition receptors (PRRs). PRRs can recognize PAMPs and initiate downstream signal activation, including the innate immune signaling cascades or programmed cell death processes [2]. Cytosolic sensors play an important role in recognizing intra-cellular PAMPs [3]. According to their structural and functional characteristics, cytosolic PRRs are mainly divided into retinoic acid inducible gene-I-like receptors (RLRs) [4], nucleotide binding oligomerization domain-like receptors (NOD-like receptors, NLRs) [5], AIM2-like receptors (ALRs) [6], cyclic GMP-AMP synthase (cGAS) [7], nucleases and other DExD/H-box family helicases besides RLRs. In the recent years, several proteins present in the nucleus also showed sensor activity, which could sense viral nucleic acid generated in the nucleus. These sensors shared distinct recognition diversities and together made up the cytosolic and nuclear innate immune sensing network.

### Cytosolic viral RNA sensing

Various kinds of pathogens, such as virus, parasites like Plasmodium and bacteria, can release RNA into the cytoplasm of infected cells [8]. RIG-I like receptors (RLRs) are a class of RNA helicases that can recognize and bind RNA from different origin, including RNA from pathogens, long non-coding RNAs, mitochondrial RNA and small interfering RNAs of cells [9]. Canonical members of RLRs include retinoic acid inducible gene I (RIG-I, also called DDX58), melanoma differentiation associated gene 5 (MDA5) and DExH-box helicase 58 (LGP2, also called DHX58) [4]. These three molecules can recognize double-stranded RNA (dsRNA) in the cytoplasm, but recognition specificity and functional characteristics are different. MDA5 and RIG-I have a similar structure, both containing the N-terminal caspase-associated recruitment domain (CARD), the helicase domain in the middle, and their respective C-terminal region [10]. N-terminal domain mediates the oligomerization of RIG-I and MDA5 and also mediates the interaction of RIG-I and MDA5 with the downstream protein mitochondrial antiviral signaling protein (MAVS). dsRNA recognition is mediated by RIG-I and MDA5 helicase domains and the C-terminal domains [11–14]. Regarding the specificity of RNA recognition, RIG-I is primarily responsible for recognizing short dsRNA with a 5′-triphosphate (5′-ppp) in the cytoplasm, while MDA5 is primarily responsible for recognizing dsRNA larger than 2000 bp such as genomic RNA of EMCV [15, 16]. Circular RNA synthesized in vitro and derived from virus can also bind and activate RIG-I [17]. Because of the constitutive expression of RLRs, the activity of RLRs needs to be suppressed at resting state to prevent excessive inflammation and the occurrence of autoimmune diseases. Structural and functional studies have revealed that RIG-I exhibits a self-inhibitory effect when not binding to dsRNA [10]. On the other hand, the combination of dsRNA can eliminate this inhibitory effect and release the CARD of RIG-I [18]. RIG-I thus oligomerizes to form the activation status. When combined with dsRNA, every four RIG-I molecules form tetramerization via CTD. RIG-I tetramerization along the dsRNA forms fiber-like aggregates, which ultimately activates downstream MAVS-mediated signaling pathways [12, 13]. Ubiquitination of RIG-I, mediated by the E3 ligases tripartite motif-containing 25 (TRIM25) and Riplet, is critical for its activation. Recent studies have revealed the relative importance of these two enzymes in the activation RIG-I. Both TRIM25 and Riplet are reported to be involved in the activation of RIG-I, but Riplet is considered to be the most important E3 ubiquitin ligase in this process [19]. The consequence of ubiquitination is: on one hand, the K63 ubiquitination of RIG-I mediated by Riplet/TRIM25 promotes the formation of fiber-like aggregation when RIG-I binds to short dsRNA. On the other hand, fiber-like aggregation formed by Riplet/TRIM25 mediated bridging of RIG-I tetramers binding with long dsRNA promotes RIG-I activation [20].

MDA5 also undergoes K63 polyubiquitination mediated activation. In cell free systems, K63 polyubiquitination stabilizes the 2CARD oligomerization of MDA5, promoting the activation of MDA5 [21]. It has been reported that TRIM65 mediates the K63 polyubiquitination of MDA5. TRIM65 catalyzes K63 polyubiquitination of MDA5 on lysine 743, which is important for the antiviral ability of MDA5 [22]. Although having a helicase domain and CTD, unlike RIG-I or MDA5, DHX58 lacks N-terminal CARDs, which prevents it from activating MAVS and downstream signals despite its dsRNA binding function [23]. Nevertheless, DHX58 can still participate in the RLR signaling pathway via regulating RIG-I and MDA5 activation. DHX58 can competitively bind dsRNA, inhibiting RIG-I activation. Meanwhile, such competition effect can protect dsRNA from Dicer mediated cleavage and promote the binding of MDA5 with dsRNA, which is important for the downstream signaling transduction [3, 24, 25].

After binding with RNA in the cytosol, MDA5 or RIG-I is recruited to outer mitochondrial membrane, where it binds MAVS via CARD to promote MAVS oligomerization. The oligomerized MAVS further recruits TRAF2, TRAF3, TRAF5 and TRAF6 to form signalosome. MAVS signalosome then recruits TBK1, providing a platform for phosphorylation of IRF3. Phosphorylated IRF3 dimerizes and translocates into the nucleus to transcriptionally activate the production of antiviral type I interferons and pro-inflammatory cytokines. MAVS can also recruit IKKα/β/γ and activate NFκB to promote various pro-inflammatory cytokines transcription [26–28].

RLRs generally exhibit background expression. However, after viral or other pathogen infection, the expression levels of MDA5 and RIG-I will be significantly increased, which indicates that there is a positive feedback mechanism for natural immune signals in the RNA receptor pathway [29, 30].

Various DExD/H box RNA helicases are reported to act as RNA sensors. DDX1-DDX21-DHX36 complex recognizes viral dsRNA in the cytosol and activates TRIF pathway [31]. DDX19A senses viral RNA and activates NLRP3 inflammasome [32]. DHX33 senses cytosolic viral dsRNA and interacts with MAVS to activate innate immune signaling in dendritic cells [33]. Moreover, DHX33 also activates the NLRP3 inflammasome after binding to dsRNA to trigger cell pyroptosis [34]. DDX60 can bind viral DNA and RNA and is essential for RIG-I activation [35, 36].

**Fig. 1:**
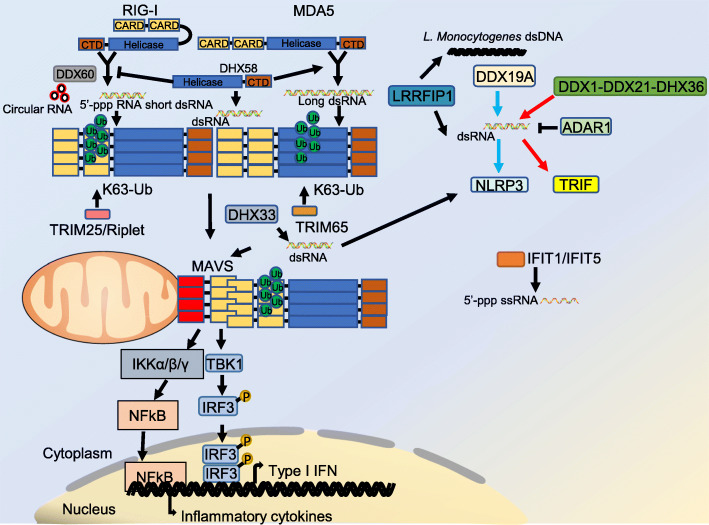
Cytosolic viral RNA sensing. The RLR family members MDA5 and RIG-I preferentially recognize long dsRNA and 5′- triphosphate short dsRNA, respectively. RIG-I can also recognize circular RNA synthesized in vitro and derived from virus. After RNA ligand binding, RIG-I and MDA5 undergo conformational changes that expose N-terminal CARDs to mediate downstream signaling. Riplet/TRIM25 or TRIM65-mediated K63-linked ubiquitination induces RIG-I and MDA5 activation, respectively. Both RIG-I and MDA5 can interact with MAVS on the outer membrane of mitochondria and activate MAVS to form signal platform, which further activates NFκB via the IKK complex or IRF3 via TBK1. DHX58 is reported to facilitate MDA5 activation, whereas it drives the inactivation of RIG-I. Besides, DDX1-DDX21-DHX36 complex recognizes viral dsRNA and activates TRIF pathway. DDX19A senses viral RNA and activates NLRP3 inflammasome. DHX33 senses cytosolic viral dsRNA and interacts with MAVS to activate innate immune signaling. DDX60 is needed for the activation of RIG-I by binding to viral RNA. ADAR1 recognizes viral dsRNA suppresses dsRNA-induced signaling. LRRFIP1 recognizes dsRNA and *L. monocytogenes* dsDNA. Moreover, IFIT1 and IFIT5 can recognize 5′- triphosphate ssRNA

Apart from the sensors raised above, several interferon-inducible sensors participate in viral RNA sensing. DsRNA sensor 2′,5′-oligoadenylate (2-5A) synthetase (OAS) catalyzes ATP to 2′,5′-oligoadenylates, which further activates ribonuclease RNase L to mediate the cleavage of viral dsRNA [37, 38]. Interferon-induced protein with tetratricopeptide repeats 5 (IFIT5) and IFIT1 recognize ssRNA carrying a 5′-triphosphate (5′-ppp) with TPR domain, protecting cells from viral infection [39, 40]. Protein kinase regulated by RNA (PKR) links cellular stress to viral sensing. PKR recognizes viral dsRNA, followed by mediating translation arrest by phosphorylating eIF2α, changing the cell translation pattern and inhibiting viral replication [41]. Moreover, PKR remains inactivated by endogenous circRNA and is activated via RNase L mediated circRNA degradation after viral infection [42]. Adenosine deaminase acting on dsRNA 1 (ADAR1) shares structural similarity with PKR for both having multiple dsRNA binding domains. Full length ADAR1 (p150) is an interferon-stimulated gene (ISG) and mainly localizes in the cytosol [43]. ADAR1 recognizes viral dsRNA and catalyzes the adenosine (A) to inosine (I), resulting in A-to-I editing. Such editing of viral dsRNA mainly suppresses dsRNA-induced signaling, including the blockage of PKR induced translation arrest and inhibition of innate immune signaling activation induced by RIG-I [44, 45]. Leucine-rich repeat (LRR) protein LRRFIP1 recognizes dsRNA and responds to VSV infection. Meanwhile, LRRFIP1 also senses dsDNA from *L. monocytogenes*. The binding of LRRFIP1 to dsRNA or dsDNA is essential for β-catenin phosphorylation. β-catenin is an important transcriptional co-activator of *Ifnb1* promoter [46] (Fig. 1).

### Cytosolic viral DNA sensing

In recent years, various DNA binding proteins have been reported to act as DNA recognition receptors. One of the most important DNA recognition receptors is cyclic GMP-AMP synthase (cGAS). cGAS is an interferon-inducible gene, which means that DNA-mediated innate immune signaling pathways also have positive feedback mechanism as RNA-mediated pathways [47]. After recognizing the double-stranded DNA (dsDNA) located in the cytosol, cGAS uses GTP and ATP to synthesize the second messenger cGAMP. Cytosolic cGAMP activates downstream STING-TBK1-IRF3 signaling. cGAS has different binding modes when recognizing DNA of different lengths. When recognizing dsDNA less than 20 bp, dimerized cGAS each binds one dsDNA nucleotide, forming a 4-molecule complex. When binding the dsDNA longer that 40 bp, the dimerized cGAS lines along the dsDNA and forms a phase-separated structure to efficiently synthesize cGAMP [48]. cGAS activated by viral DNA can induce the activation of innate immunity and up-regulate the expression of type I interferon and various pro-inflammatory cytokines, subsequently activating adaptive immune system to promote the elimination of pathogens [49]. Recently, it has been reported that via N-terminal phosphoinositol binding domain, cGAS can localize to phagocytes plasmalemma. N-terminal domain of cGAS selectively interacts with PI (4,5) P2, mutation of this domain results in cGAS lipid binding defects and mislocates to the cytoplasm and nucleus. The mis-localized cGAS causes strong interferon in response to genotoxic stress, but the cGAS response induced by viral infection is weakened [50].

Several members of DExD/H helicases are reported to respond to cytosolic dsDNA. It has been reported that both DHX9 and DHX36 can sense cytosolic CpG-DNA, with DHX9 recognizing CpG-B DNA with DUF1605 domain and DHX36 recognizing CpG-A DNA with DEAH domain [51]. DDX41 is the first reported cytoplasmic DNA sensor. DDX41 uses its DEAD domain to recognize dsDNA and interact with STING to activate the innate immune signaling [52]. RNA Pol III is the sensor for AT-rich dsDNA in the cytoplasm and is widely expressed. RNA Pol III recognizes viral dsDNA and transcribes it into dsRNA, which in turn recognized by RIG-I to activate innate immune response [53]. The DNA-dependent protein kinase (DNA-PK), a DNA damage response mediator, was reported to act as a dsDNA sensor. DNA-PK is a signaling complex consisting of three members, namely the catalytic subunit DNA-PKcs and DNA binding subunits Ku70 and Ku80 [54]. DNA-PK recognizes cytoplasmic dsDNA of vaccinia virus, which is important for IRF3-mediated innate immune responses in fibroblasts [55]. Moreover, DNA-PK relies on STING, TBK1, and IRF3 to induce the production of cytokines during VACV infection [56]. It is well known that three prime repair exonuclease 1 (TREX1) can protect host from excessive inflammation via efficiently degrading cytosolic endogenous DNA [56, 57]. Moreover, in the case of HIV infection, inhibiting the function of TREX1 can augment the production of type I IFN and suppress HIV replication and spreading, indicating that TREX1 negatively regulates HIV-induced antiviral innate immunity [58].

**Fig. 2:**
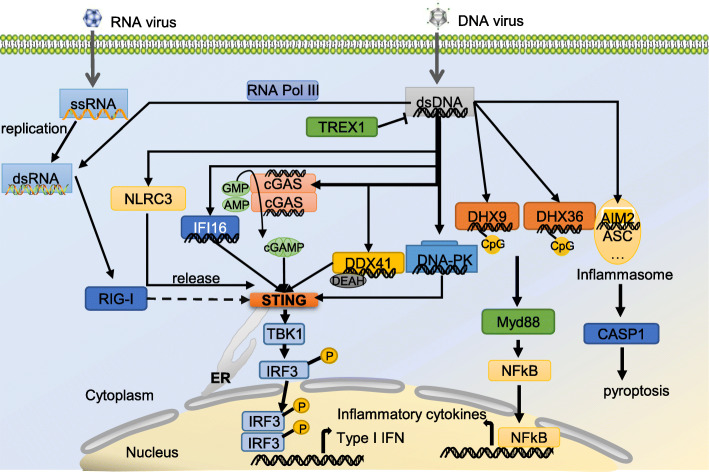
Cytosolic viral DNA sensing. DNA is a PAMP that can be delivered to the cytoplasm of host cells during microbial infection. Several DNA sensors have been reported to promote the activation of innate immune signaling by activating a STING-dependent signaling pathway. Among them, cGAS can catalyze ATP and GTP to generate the second messenger cyclic GMP-AMP (cGAMP) and then activate STING. However, the molecular basis by which other DNA sensors activate STING is not well understood. RNA polymerase III can activate the immune response in a STING-independent way, RNA polymerase III can transcribe poly (dA-dT) into dsRNA, which is then recognized by RIG-I. Several members of DExD/H helicases are also respond to cytosolic dsDNA, such as DHX9, DHX36 and DDX41. In IRF3-mediated innate immune responses of fibroblasts, DNA-PK recognizes cytoplasmic dsDNA of vaccinia virus. TREX1 is well known to protect host from excessive inflammation via efficiently degrading cytosolic endogenous DNA. PYHIN family proteins: AIM2 and IFI16. AIM2 senses dsDNA from virus and oligomerizes to form AIM2 inflammasome. IFI16 binds to cytoplasmic viral DNA and mediates the activation of STING-TBK1-IRF3 axis. Moreover, NLRC3 recognizes dsDNA and releases STING, which activates type I interferon induction

Another family of cytosolic DNA sensor are PYHIN family proteins. Members of this family contain an N-terminal Pyrin domain, which mediates the interaction with other Pyrin domain containing proteins. PYHIN family members also contain one or two C-terminal HIN domains, which are involved in DNA binding [59, 60]. Human genome contains 4 PYHIN family members, whereas murine genome contains 14 members [61]. Of the PYHIN members, AIM2-like receptors (ALRs), including AIM2, IFI16 and IFI204 (ortholog of human IFI16) are identified as PRRs [6]. Unlike IFI16 or IFI204, AIM2 exists in both human and murine genome. AIM2 exhibits a self-inhibitory state without binding with DNA, which is first thought to be mediated by the self-Pyrin-HIN domain interaction [62]. However, a recent study suggested that Pyrin domain was not self-inhibitory, but provided a platform for ligand binding [63]. AIM2 senses dsDNA from virus such as vaccinia virus and forms AIM2 inflammasomes along with caspase-1 and apoptosis-associated speck-like protein containing CARD (ASC) [64]. IFI16 was reported to bind viral DNA of herpes simplex virus 1 (HSV-1) in the cytoplasm, then mediates the activation of STING-TBK1-IRF3 axis [65]. However, as an ortholog of human IFI16, IFI204 plays controversial roles in innate immune response. IFI204 is important for TLR4 signaling and may facilitate host to fight against *Staphylococcus aureus* and *Francisella* infection, but negatively regulates antiviral innate immunity via inhibiting the target promoter binding of IRF7 in the nucleus [66–69]. Exploring the function of other PYHIN family members, especially their roles in antiviral innate immunity, will help understand the compilated regulation mechanism of innate immune response. Moreover, NLRC3 can act as a DNA sensor. NLRC3 recognizes dsDNA from HSV-1, releasing STING from binding to NLRC3, which is an inactive form of STING, to activate type I interferon induction [70] (Fig. 2). In order to link the DNA/RNA sensors with recognition of viruses, we list the viruses that are sensed by the individual PRRs in Table 1.

**Table 1:** Pathogens and corresponding receptors in cells

Receptor	Pathogen	References
RIG-I	Sendai virus	[71]
	Newcastle disease virus	[71]
	respiratory syncytial virus	[72]
	measles	[73]
	Nipah	[74]
	vesicular stomatitis virus	[71]
	rabies virus	[75]
	influenza A/B	[72, 76]
	Ebola	[74]
	hepatitis C virus	[77]
	murine hepatitis virus	[78]
MDA5	encephalomyocarditis virus	[79]
	Theiler’s virus	[76]
	vaccinia virus	[80]
MDA5 and RIG-I	Japanese encephalitis virus	[76]
	dengue virus	[72]
	West Nile virus	[81]
RNA pol III	EBV	[53]
IFI16	HSV-1	[65]
DDX41	HSV-1, *L. monocyte genes*, adenovirus	[52, 82]
DNA-PK	MVA, HSV-1	[55, 83]
cGAS	HSV-1	[7]
LRRFIP1	*L. monocyto genes*, VSV	[84]

### Nuclear viral DNA/RNA sensing

Virus, including DNA and some RNA virus, replication generates nucleic acid in the nucleus. Generally, most DNA virus replicate within the host cell nucleus, which leads to their genomic DNA ejection or viral DNA/RNA generation during their replication in host cell nucleus [85]. Most RNA viruses replicate in the cytosol. However, retroviruses and some single-stranded RNA virus, such as influenza virus, can replicate in the nucleus and their genomic RNA can be detected in the nucleus of the infected cells. Most cytoplasmic replicated RNA viruses, such as VSV or Rabies virus, can generate the leader RNA transcript which can present in host cell nucleus and mediate viral replication and transcription.

Most PRRs are localized on the plasma membrane, endosomes or cytoplasm. Recent evidence suggested that several proteins located in the nucleus could also act as viral sensors. For example, IFI16 can recognize HSV-1 virus DNA within the nucleus and leads to the expression of IFNβ via activation of IRF3 signaling [86]. Nuclear IFI16 can also recognize viral DNA from HIV, causing the pyroptosis of infected CD4^+^ T cells [87]. When human endothelial cells are infected with KSHV, IFI16 can interact with procaspase-1 and ASC to form functional inflammasome. This inflammasome is involved in viral DNA recognition, caspase-1 activation and IL-1β maturation [88].

cGAS is widely accepted as a cytoplasmic dsDNA sensor. Recently several researches suggested that cGAS is primarily a nuclear protein and the tethering prevents genomic DNA from activating cGAS. Nuclear localized cGAS is tightly tethered through salt-resistant interaction. cGAS tethering requires intact nuclear chromatin to keep cGAS in resting state and prevent its own reactivity [89]. In addition, the nucleosome-binding interface occupies the strong dsDNA binding surface on cGAS, thus effectively prevents cGAS from oligomerizing into a functional active state [90]. Lately, cGAS is reported to bind to nuclear IFI16 and can promote the stability of IFI16 in human fibroblasts and keratinocytes during HSV-1 infection [91].

Normally, hnRNPA2B1 is an RNA-binding protein localized in the nucleus. Cao et al. found that hnRNPA2B1 could bind viral DNA in various mouse and human cells nuclei during HSV-1 infection [92–94]. hnRNPA2B1 can form a complex with viral DNA and undergo homodimerization and demethylation, resulting in the translocation of the complex into the cytoplasm and the activation of immune response mediated by type I interferon signaling.

Our group confirmed that scaffold attachment factor A (SAFA) localized in the nucleus can act as a trans-activator of antiviral genes and a viral dsRNA sensor. Upon viral infection, SAFA senses dsRNA generated from virus (such as HSV-1 or VSV) replication, oligomerizes, and facilitates antiviral immunity inducing IFNβ production through interacting with DNA topoisomerase 1 (TOP1) and SWI/SNF-related matrix-associated actin-dependent regulator of chromatin subfamily A member 5 (SMARCA5) in human and mouse primary cells. Besides, oligomerized SAFA mediates IFNB1 transcription and maintains antiviral status of infected cells through interacting with enhancers and super-enhancers [95].

NONO (non-POU domain containing octamer binding) is an important sensor for HIV capsid recognition in the nuclei of dendritic cells and macrophages. The directly binding affinity of NONO to weakly pathogenic HIV-2 capsid is stronger than that of highly pathogenic HIV-1. NONO is necessary for cGAS to associate with nuclear HIV DNA and thus activates cGAS. Moreover, NONO can recognize conserved regions in the capsid of HIV and has limited escape tolerance to mutations. The promotion of DNA sensing by cGAS followed by detection of nuclear viral capsid by NONO reveals the basic strategy of differentiating non-self from self in the nucleus [96].

It is well known that hexamethylene bis-acetamide-inducible protein 1 (HEXIM1) inhibits the positive transcription elongation factor b (P-TEFb) and can bind to RNA in the nucleus [97]. P-TEFb controls transcriptional elongation through RNA polymerase II. HEXIM1 is the crucial component of the 7SK RNP complex and plays a significant role in inhibiting RNA polymerase II phosphorylation and succeeding transcriptional elongation [98–101]. HEXIM1 plays an critical role in regulating the innate immune response mediated by DNA infection, it can bind long non-coding RNA such as NEAT1 to promote the formation of the HDP-RNP complex, which acts as a platform for activation through the cGAS-STING pathway and subsequent IRF3 phosphorylation [102].

More and more proteins have been found to recognize nuclear viral nucleic acids, which raises question of how they distinguish non-self from self-nucleic acids substances. It may be partially explained by the highly ordered structure of the nucleus and far more research is needed.

## The strategies of viral innate immune evasion

Viruses have evolved a series of effective tactics to evade the surveillance of host’s innate immunity in order to replicate and spread efficiently. We thus describe the molecular strategies that may be used by viruses to evade host innate immunity, including viral evasion of PRRs detection and block the activation of signaling molecule at different levels.

PRRs function as sensors to initially response to invading pathogens. Nevertheless, viruses have developed different strategies to evade PRRs surveillance. Viruses can utilize cellular membrane to form a confined space or replicate in organelles (endoplasmic reticulum, Golgi complex or mitochondrion) to avoid being detected by RLRs. For example, Dengue virus (DENV) can replicate in endoplasmic reticulum to efficiently hide viral dsRNA from the cytoplasm [103]. Several viruses can modify their genomes to avoid being detected by RLRs. In order to escape the surveillance of RIG-I, Borna disease virus (BDV) encodes phosphatases that convert 5′-triphosphate (5′-ppp) on its genome to 5′-monophosphate (5′-p) [104]. In addition, many viruses can block PRRs recognition of viral nucleic acids by using host-encoded or viral proteins. For example, EBOV’s viral protein 35 (VP35) competitively binds to viral dsRNA to prevent dsRNA from being detected by RIG-I [105].

In addition to developing mechanisms to evade host detection, viruses also target the PRRs level by segregating and modifying the receptors. HSV-1 capsid protein US11 can interact with RIG-I and prevent RIG-I from forming RIG-I/MAVS complex [106]. Lys63 linked polyubiquitylation is important for the activation of RIG-I. The NS1 protein of Influenza A virus (IAV) can inhibit the activity of the E3 ubiquitin ligases TRIM25 or Riplet and thus prevent Lys63 linked ubiquitination of RIG-I [107, 108]. MDA5 is an intracellular sensor that can recognize viral long dsRNA [10, 109]. The V protein of Parainfluenza virus 5 (PIV5-V) can recognize and bind to MDA5 structural motif, disrupting the ATP hydrolysis activity and filament formation of MDA5 [110]. In the absence of viral infection, phosphorylation of threonine or serine leaves MDA5 and RIG-I in an inactive state. In the case of virus infection, PP1α or PP1γ mediated dephosphorylation is crucial for RIG-I and MDA5 activation. The V proteins of Nipah virus (NiV-V) can act as an antagonistic to PP1γ and PP1α, reducing the dephosphorylation of MDA5 and RIG-I, impairing downstream innate immune responses [111].

Degradation of the sensor or the key component is an effective way to inhibit RLR signaling. Intriguingly, many viruses can directly degrade RLRs by expressing proteases. For example, 3C protease of Enterovirus 71 (EV71), Poliovirus, EMCV, Echovirus, Rhinovirus type 1A and Rhinovirus type 16 can directly cleave RIG-I [112], whereas the 2A protease of EV71 can cleave MDA5 [113].

**Fig. 3:**
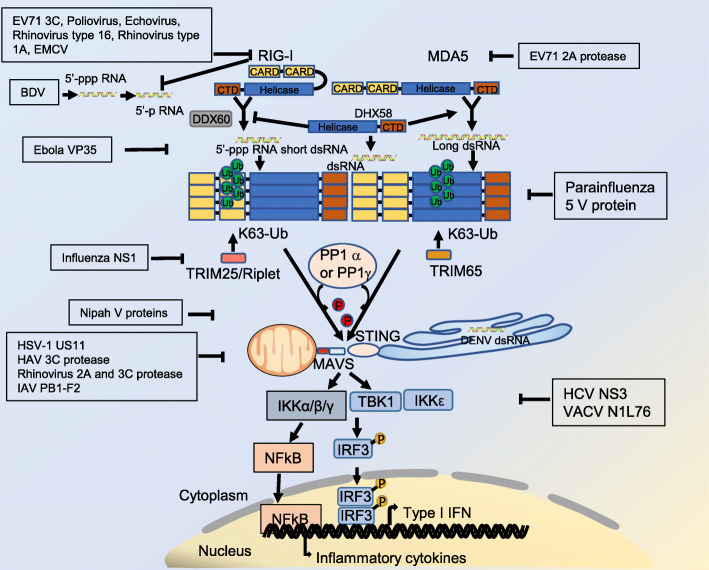
Innate immune evasion strategies of targeting RLR signaling pathway**.** Viruses can escape from host immune clearance. For example, DENV can replicate in endoplasmic reticulum to efficiently hide dsRNA from the cytoplasm. BDV encodes phosphatases to process the 5′-ppp on its genome to 5′-p. The protein viral protein 35 (VP35) of EBOV can interact with dsRNA to prevent dsRNA from being detected by RLRs. Moreover, RLR signaling can be inhibited by viral proteins that either directly bind MDA5, RIG-I, MAVS, TBK1 to inhibit their function or induce their degradation

The signal platform formed by MAVS is critical for activating the downstream molecules TBK1 (TANK binding kinase 1) and IRF3 (interferon regulatory factor 3). Degradation of the adaptor MAVS is also a common event during virus infection. 3C protease from hepatitis A virus (HAV), 2A protease and 3C protease from rhinovirus can directly target and cleave MAVS [114–117]. Interestingly, IAV can block MAVS signaling activation in a proteasome-independent manner. The IAV protein PB1-F2 can bind to MAVS transmembrane region to block type I interferon signaling by decreasing the mitochondrial localized MAVS protein level [118]. In the innate immune response, TBK1 is an important kinase. When the activity of TBK1 is blocked, IRF3 cannot be phosphorylated and cannot be dimerized or translocate into nucleus. The NS3 protein of hepatitis C virus (HCV) and the N1L76 protein of vaccinia virus (VACV) can bind to TBK1 and inhibit the downstream signaling activation [119, 120] (Fig. 3).

cGAS acts as a crucial DNA sensor for the detection of viral dsDNA and mediates the production of proinflammatory cytokines and IFNs. Viruses are able to inhibit cGAS dependent innate immune signaling via distinct approaches. Viral proteins can bind to cGAS and affect its DNA-binding activity or enzymatic activity. For example, ORF52 of Kaposi’s sarcoma-associated herpesvirus (KSHV) can interact with cGAS, blocking enzymatic activity of cGAS and impairing downstream innate immune responses, ultimately facilitating the evasion of KSHV [121]. Viral proteins can also mediate the degradation of cGAS. After DENV infection, cGAS can detect the released DNA from the mitochondria. Nevertheless, NS2B protein of DENV can degrade cGAS by lysosomal degradation [122]. IFI16 is the nuclear DNA sensor. The viral E3 ubiquitin ligase ICP0 of HSV-1 can medicate proteasomal degradation of IFI16 [123]. pUL83 protein of the human cytomegalovirus (HCMV) binds to PYD domain of IFI16 and inhibits IFI16 oligomerization, dampening downstream immune signaling activation. Besides, VACV proteins C16 and C4 can antagonize DNA-PK. C4 and C16 can bind to Ku and prevent Ku from binding to DNA [124, 125], thus reducing the production of chemokines and cytokines, decreasing the recruitment of inflammatory cells, inhibiting the IRF3 activation. Moreover, E1A oncoprotein of human adenovirus 5 and the ICP0 protein of HSV-1 can also block DNA-PK induced robust and broad antiviral response [56].

**Fig. 4:**
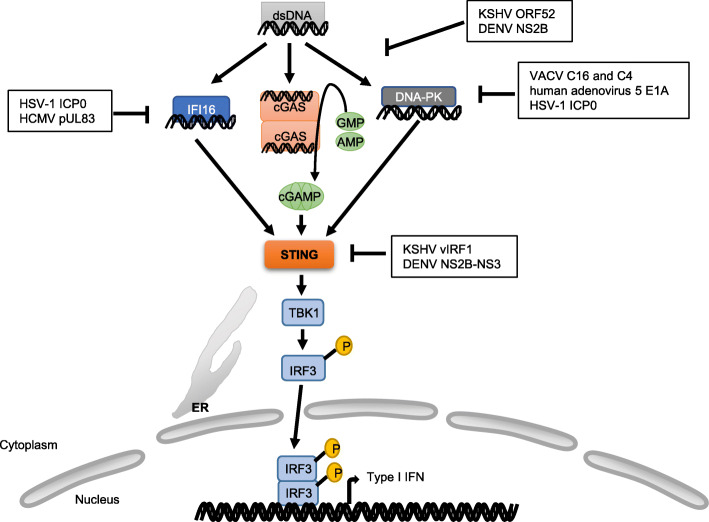
Innate immune evasion strategies of targeting DNA signaling pathway. DNA signaling can be restrained by viral proteins that either interact with IFI16, cGAS, DNA-PK or STING directly or cause their degradation

It has been reported that viruses can disturb STING’s function. For example, viral protein vIRF1 from KSHV can bind to STING as an antagonist [121]. In addition, the protease complex NS2B–NS3 of DENV can cleave STING to reduce the IFNs signaling transduction [126] (Fig. 4).

## Conclusions

Research over the past two decades have extensively investigated the role of innate immune system and related PRR in virus recognition and initiation of antiviral defenses. Recent studies found viral RNA and DNA sensing in the nucleus of viruses infected cells. The detail regulatory mechanism of nuclear viral recognition and subsequent epigenetic alteration await to be investigated. Besides, viruses have developed diverse strategies to suppress immune responses for evasion. However, The research of immune escape lags far behind immune recognition. Far more research is needed to broadly clarify the mechanisms and identify the potential targets for viral immune evasion. Given the initial promising results, a better understanding of host-viral biology will bring great benefits to the novel clinical applications.

## Data Availability

Not applicable.
